# Composite Behavior of Nanopore Array Large Memristors

**DOI:** 10.3390/mi16080882

**Published:** 2025-07-29

**Authors:** Ian Reistroffer, Jaden Tolbert, Jeffrey Osterberg, Pingshan Wang

**Affiliations:** 1 Holcombe Department of Electrical and Computer Engineering, Clemson University, Clemson, SC 29634, USA; ianreistroffer0@gmail.com (I.R.); tolber3@g.clemson.edu (J.T.); jeff_osterberg@hotmail.com (J.O.); 2 Department of Physics, South Dakota School of Mines and Technology, Rapid City, SD 57701, USA; 3 Sandia National Laboratories, Albuquerque, NM 87123, USA

**Keywords:** nanofluidics, memristors, rectification, membranes, nanopores

## Abstract

Synthetic nanopores were recently demonstrated with memristive and nonlinear voltage-current behaviors, akin to ion channels in a cell membrane. Such ionic devices are considered a promising candidate for the development of brain-inspired neuromorphic computing techniques. In this work, we show the composite behavior of nanopore-array large memristors, formed with different membrane materials, pore sizes, electrolytes, and device arrangements. Anodic aluminum oxide (AAO) membranes with 5 nm and 20 nm diameter pores and track-etched polycarbonate (PCTE) membranes with 10 nm diameter pores are tested and shown to demonstrate memristive and nonlinear behaviors with approximately 10^7^–10^10^ pores in parallel when electrolyte concentration across the membranes is asymmetric. Ion diffusion through the large number of channels induces time-dependent electrolyte asymmetry that drives the system through different memristive states. The behaviors of series composite memristors with different configurations are also presented. In addition to helping understand fluidic devices and circuits for neuromorphic computing, the results also shed light on the development of field-assisted ion-selection-membrane filtration techniques as well as the investigations of large neurons and giant synapses. Further work is needed to de-embed parasitic components of the measurement setup to obtain intrinsic large memristor properties.

## 1. Introduction

Brain-inspired neuromorphic computing is promising to address the growing energy and capacity problems of current digital computing techniques [1] by developing hardware-based neural networks [2]. As a result, dynamic and complex memristors (memory resistors) that emulate the functions of synapses and neurons are critical. Such devices were hypothesized in the Hodgkin–Huxley neuron model [3] and have been both theorized [4] and demonstrated [5], albeit questioned [6]. The memristor is considered the fourth fundamental passive circuit component, owing to its unique ability to vary in resistance under an applied voltage [4]. They have been realized for a variety of important applications, such as memory circuits, logic operations, neural networks, and more [7]. A variety of physical phenomena in solid-state inorganic [1] and organic [8] materials have been exploited to build memristors, achieving rapid progress in the field [9]. For instance, both inorganic and organic memristors have reported an energy efficiency close to that of biological synapses [10,11]. Further, the tunability of the behavior of such memristors has been demonstrated through manipulations of filament sizes (in the case of Ag-dendrite-based devices) [12], channel length [13], and electrode size [14], the latter two of which are uniquely important to this study where control of the functional layer dimensions is key. However, inorganic solid-state memristors lack compatibility with biosystems and the capability to recognize chemical signals. Reducing device-to-device and cycle-to-cycle variations remains challenging. Organic solid-state memristors [15] usually suffer from large footprints [8] and are incompatible with CMOS fabrication processes. Moreover, the Set and Reset processes commonly take place in destructive fashions due to the small device sizes [16]. Thus, endurance and stability are significant concerns [8].

**Figure 1:**
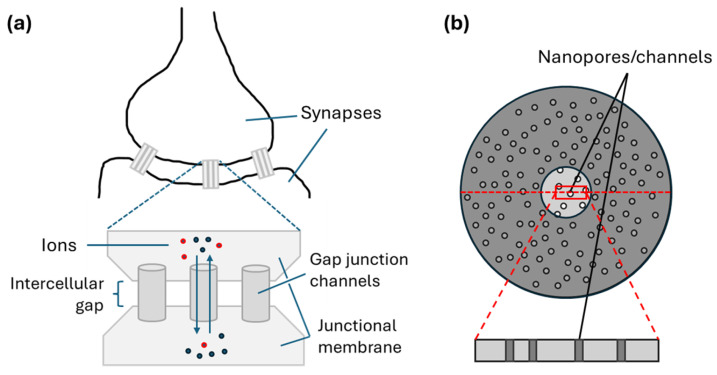
(**a**) Electrical synapses with gap junction channels allowing a direct communication between the cytoplasm of the two coupled cells via anions (red) and cations (black) [17]. (**b**) A nanoporous membrane with many channels in parallel. The number of active pores can be reduced by blocking membrane surface, such as the dark grey areas. The inset shows the nanochannels from a slice cross-section of the membrane.

Inspired by ion channels on cell membranes (Figure 1a) [17], fluidic memristors [18] with nanometer [19,20] or angstrom [20,21,22] critical dimensions have been recently proposed as an alternative approach to help address the problems [23] of biocompatibility, ion selection [24] (which may enable new neuromorphic functionalities), and device-to-device and cycle-to-cycle variations. Artificial nanopores [25], nanochannels, nano-slits [21,22], and various channel surface treatments [26] have been exploited to demonstrate fluidic memristive and synaptic behaviors with different ionic electrolytes, such as KCl, NaCl, and CaCl_2_ [25]. Asymmetric electrolyte concentration [27,28,29], device geometry (e.g., conical pores [20] and tapered nanochannels [30]), and surface charge distribution [26], or the combination of these factors [25], have been shown to induce and control ion transport for tailored memristive performance. Energy efficiency comparable with the energy consumption of biological synapses (a 2–23 fJ/spike per angstrom-channel [31]) has been reported. These efforts mostly involve a single [20] or a few [25] nanofluidic structures. They are essential to elucidate the fundamentals, including ion transport and memristive behavior [19,20,21,22].

So far, memristor development [8] has been primarily guided by solving static and small-scale problems, such as crossbar arrays for multiply-and-accumulate (MAC) or simple pattern recognition [1]. There have been few publications of memristor or memristor-based neurons for dynamic learning [32] or large-scale problem solving. For instance, Purkinje cells receive all incoming sensory and motor information and generate the sole output of the cerebellar cortex, critical for motor learning, error correction, and rapid responses [33,34]. These neuron cells [35,36] usually have more than 100,000 synapses. Playing a similar role are pyramidal neurons [37,38], which can have millions of synapses [39,40]. At the same time, an extra-large synapse can have hundreds of ion channels (Figure 1a) [41,42,43]. Thus, millions of ion channels may operate in parallel in such neurons, as a neural network.

Full-scale digital neuromorphic modelling of such neural networks is difficult [34,44,45]. Therefore, the behavior of extra-large fluidic composite memristors, such as millions of nanopores in parallel (Figure 1b), is of interest. Also of interest is the behavior of large fluidic memristors in series, e.g., for reservoir computing [46]. Such composite memristor configurations have been theoretically analyzed [47,48,49] but not experimentally investigated. Moreover, currently reported fluidic memristors were synthesized in specialized chemistry or material labs [19,21,22]. Corresponding electrical measurements usually require shielding [25]. In this work, we report the electrical behavior of extra-large composite fluidic memristors of different materials and pore sizes. Commercially available nanopore membranes are used, and no shielding is needed.

## 2. Materials and Methods

**Figure 2:**
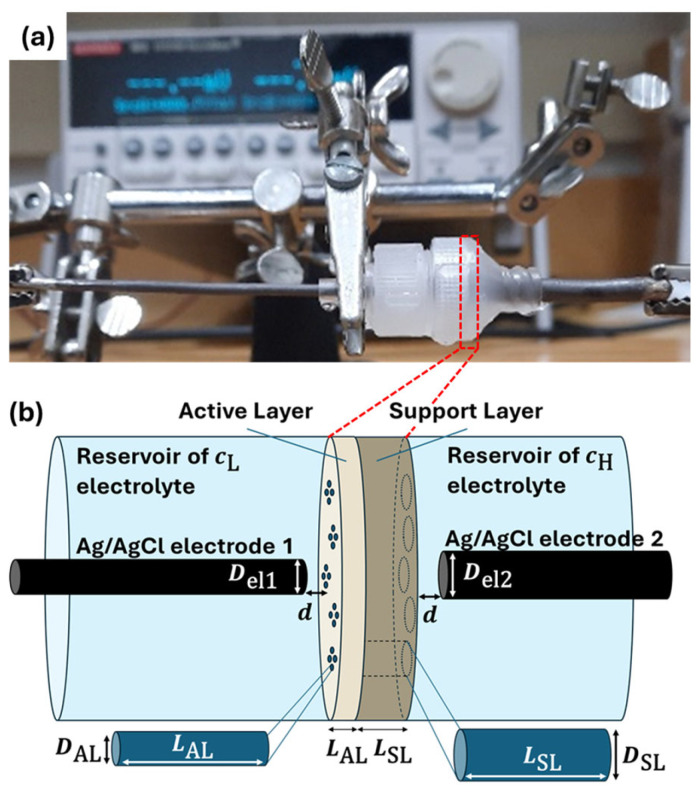
(**a**) A photograph of the experimental setup. A SourceMeter supplies a voltage to the Ag/AgCl electrode on the left-hand side and measures the current through the electrode on the right-hand side. (**b**) A schematic (not to scale) of the components inside the membrane holder. Both electrode faces are positioned a distance *d* (about 10 μm) from either side of the membrane. In the case of PCTE and AAO isotropic membranes, only a single layer (AL) is present; in the case of AAO anisotropic membranes, an additional layer (SL) is present with its own unique width, pore diameter, and pore density. The volume of the *c_L_* and c*_H_* solutions are 0.79 and 0.3 mL, respectively.

Figure 2a is the experimental system. Reproducible measurement is critical but challenging for the study of ion transport in electrolytes (e.g., in biosensors [50].) This is especially true for the composite memristors in Figure 2a, where liquid electrolytes are confined in space and ion diffusions through a large number of nanopores can quickly depolarize ion concentration, which is a unique issue for this work [50]. Therefore, rapid system operation is essential.

Figure 2b is a schematic of the membrane-based composite memristor. It consists of a membrane, hosting large arrays of nanopores/channels in parallel, that separates two reservoirs of electrolyte solution. One reservoir contains a low-concentration (*c_L_*) electrolyte, which is always set to 0.1 mM in this study, and the other contains a high concentration (*c_H_*), which may be 0.1, 1, 10, or 100 mM. Two Ag/AgCl electrodes [51] of diameters *D*_el1_ = 2 mm and *D*_el2_ = 4 mm, suspended by high-resolution positioners, are submerged in either reservoir to supply an electric voltage of either ±100 mV or ±1 V across the system. A compliance current of 3 A was used in all measurements. The voltage is delivered as a triangle waveform with a sweeping frequency of 0.01, 0.1, or 1 Hz by a Keithley 2612 SourceMeter. For system characterization, electrochemical impedance spectroscopy (EIS) measurements were made using a Digilent Analog Discovery Impedance Analyzer 2 (AD2) sourced from Digilent, Inc., Austin, TX, USA (Section S1). The software Keithley Test Script Builder (version KTS-850J05) was used to program and read data from the SourceMeter, and the software WaveForms (version 3.19.5) was used to program the AD2 and analyze impedance data. The setup enables rapid electrolyte refreshing as well as rapid and accurate tuning of electrode-to-membrane surface distance *d* for reproducible measurements. Nevertheless, manual operations remain a source of measurement uncertainty.

**Figure 3:**
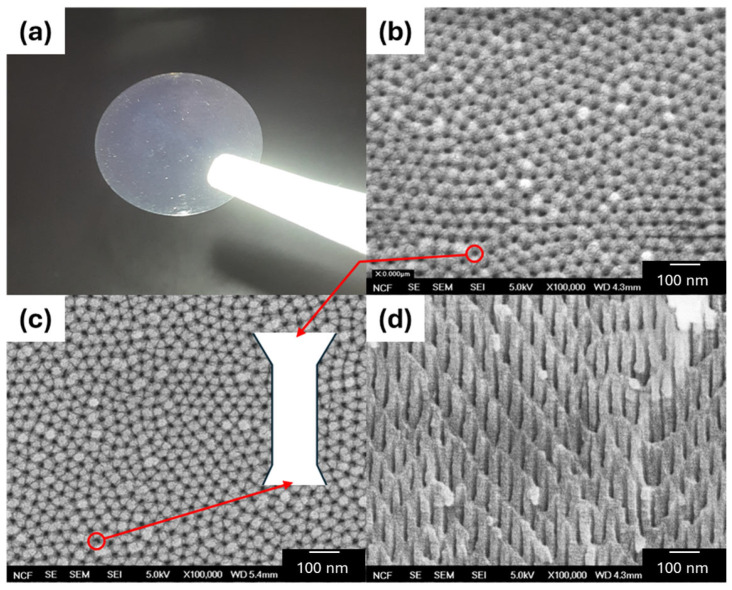
(**a**) A photograph of the 13 mm diameter AAO isotropic membrane held by tweezers. (**b**–**d**) SEM scans of the membrane showing (**b**) the top surface (20 nm diameter pores/channels), (**c**) the bottom surface, and (**d**) a cross-section slice (all used with permission by InRedox, Longmont, CO, USA). The white inset diagram in (**c**) illustrates a potential slight asymmetry of the top and bottom conical openings due to tapering.

Three different membrane types were tested: PCTE and isotropic AAO (both containing only a single “active” layer (AL)) and anisotropic AAO (multi-layered, containing both an AL and a “support” layer (SL)). For the purposes of this study, the AL and SL are treated identically, only differing in their porosity and pore dimensions (see Table 1). Figure 3a shows a picture of the AAO isotropic membrane with 20 nm pore diameter, and Figure 3b–d shows SEM scans of it from various orientations. Each membrane is 13 mm in diameter with an effective diameter of 9 mm when assembled in the membrane holder (due to tight surface blockage by an annular shelf inside the holder, which prevents the membrane from moving). Hence, we only expect concentration mixing to occur across the inner 9 mm diameter membrane interface. We further approximate that the number of effective pores corresponds to those normal to the cross-section of the 2 mm diameter electrode *D*_el1_ (discussed further in Section S1). The membrane and pore/channel dimensions for each type are summarized in Table 1.

Three different electrolyte species, KCl, NaCl, and CaCl_2_, were tested, which are commonly used in nanofluidic experiments [22]. A comparison between the salt types was anticipated to provide insight into how different overall ion mobilities will affect the memristive and ionic current rectification (ICR) behavior (Figures S4 and S7, Supporting Information). The electrical properties of the key components, i.e., the bulk electrolytes [52] and membrane pores, need to be characterized and modelled [53] for the study of memristive phenomena. Thus, the parameters of the system, including the high/low electrolyte concentration ratio, electrode–membrane separation distances (*d*), and sweeping voltage amplitudes and frequencies, are examined to identify operating conditions where the nanopore behaviors dominate parasitic effects with less measurement uncertainties.

**Figure 4:**
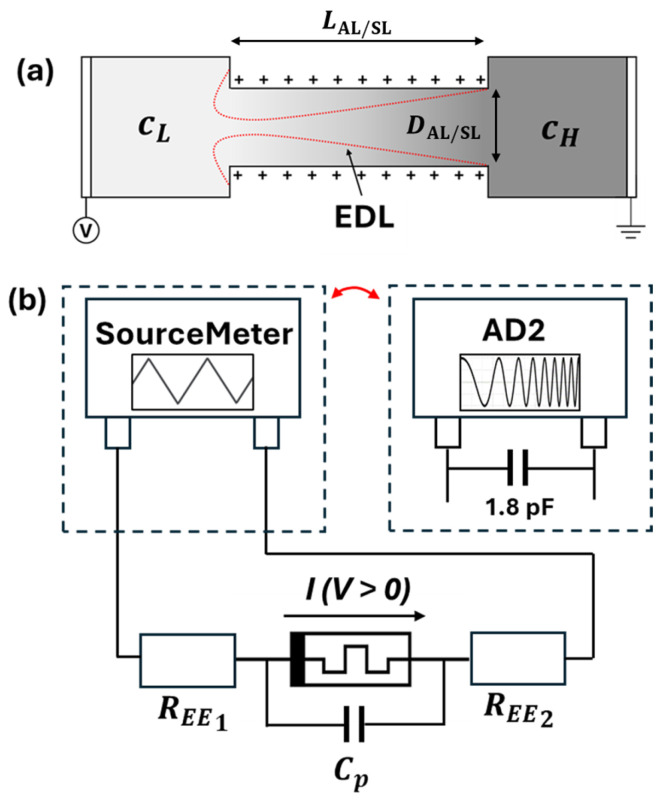
(**a**) Schematic representation of a single nanochannel connecting reservoirs of *c_L_* solution (light grey, low concentration) and *c_H_* solution (dark grey, high concentration). The EDL is represented in the red enclosure, corresponding to an enrichment of anions that have migrated toward the positively charged channel walls. (**b**) A diagram of the electrical measurement circuit. Depending on the measurement, either the SourceMeter or AD2 device may be connected to the system. Using the AD2, a parasitic capacitance of 1.8 pF is in parallel with the system. *C_p_* is parasitic capacitance across the nanopore array. *R_EE_* is the resistance of the electrode–electrolyte interfaces.

The degree to which ionic current is rectified across the nanochannel is dependent on the electric double layer (EDL), a region of electrical potential resulting from fixed surface charges on the walls of the channel [54,55,56]. A schematic representation of the EDL in a single nanochannel is shown in Figure 4a, with possible dimensions ($L_{AL/SL}$ and $D_{AL/SL}$) in this study listed in Table 1. ICR originates from the difference in EDL screening along the nanochannel, creating areas of high and low ion conductivity and leading to an accumulation or depletion of ions in the channel based on the applied potential polarity [57]. The surface charge density *σ*_s_ responsible for establishing the EDL can be determined through conductance measurements using various symmetric electrolyte concentrations (without a bulk concentration gradient) [57,58,59]. This and the mechanism governing ICR are discussed in further detail in Section S1.

## 3. Results

**Figure 5:**
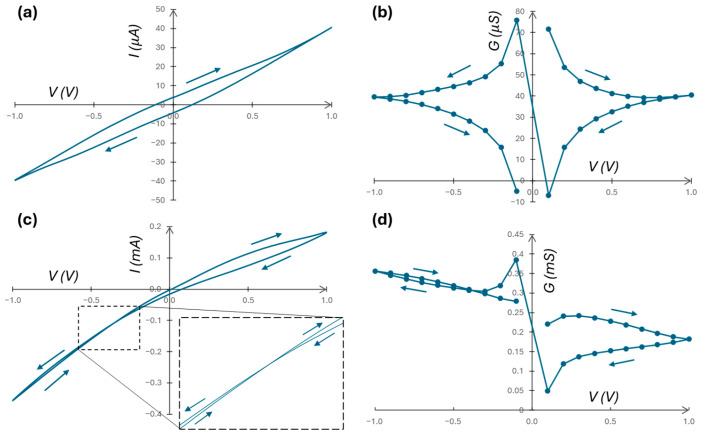
(**a**,**c**) Typical *I*/*V* measurements and (**b**,**d**) their associated *G*/*V* measurements using an AAO Isotropic membrane (with pore size of 20 nm and 50-μm channel length), applying a 1 V amplitude signal with 0.1 Hz frequency, using KCl electrolyte. KCl concentrations are (**a**,**b**) *c_L_* = *c_H_* = 0.1 mM and (**c**,**d**) *c_L_* = 0.1 mM and *c_H_* = 100 mM. The inset in (**c**) shows the memristive self-crossing point. In all figures, arrows indicate scan direction.

The composite memristor system in Figure 2 is measured using various electrolyte species, *c_H_* concentrations, applied potential amplitudes and frequencies, and membrane types. A triangle staircase *V* over 40 steps is applied to the electrode submerged in *c_L_* solution for 2 consecutive cycles, with a maximum/minimum value of +1/−1 V unless noted otherwise for special cases. The output current is measured at the end of each step (Section S2). Figure 5a,b shows the *I*/*V* and *G*/*V* relationships when *c_H_* = *c_L_* = 0.1 mM. The results are typical for an electrical double-layer capacitor [50], i.e., *C_p_* in Figure 4b, which represents the composite capacitor in parallel with the nanopore array. *C_p_* involves two double-layer capacitors associated with the two surfaces of the membrane and the capacitance of the membrane. The symmetric *I*/*V* curve indicates negligible asymmetric pore openings (see the Figure 3c inset). When *c_H_* > *c_L_*, asymmetric and memristive behaviors are induced. Figure 5c shows a typical *I*/*V* relationship, which is self-crossing (type I) [60] memristive behavior, and Figure 5d shows the corresponding *G*/*V* relationship [60]. Nevertheless, the hysteresis loop is not symmetric, and the cross-point is offset from the origin (*I* = 0, *V* = 0). A possible source for the asymmetric loop and offset cross-point is the coupling between the positive capacitance *C_p_* in Figure 4b and the pore-array memristor, similar to *C*_1_*M*_2_ coupling described and simulated in [61] (p. 6462) by the combined *I*/*V* response of a capacitive and memristive element connected in parallel. We expect the surface charge of the channel walls to govern the phenomenon, i.e., that the potential at the point of crossing is the component of the surface electric field in the direction of ion transport [19,62]. Furthermore, Figure 5c shows significant differences between forward (*I_F_*) and reverse (*I_R_*) currents for the same applied voltage values, i.e., a nonlinear behavior different from those observed in [61]. The difference is likely due to the lack of nonlinearity and membrane potential (further discussed below) consideration in the memristive components in [61]. There is no indication of inductive (negative capacitance) behavior in Figure 5c *I*/*V* measurements.

**Figure 6:**
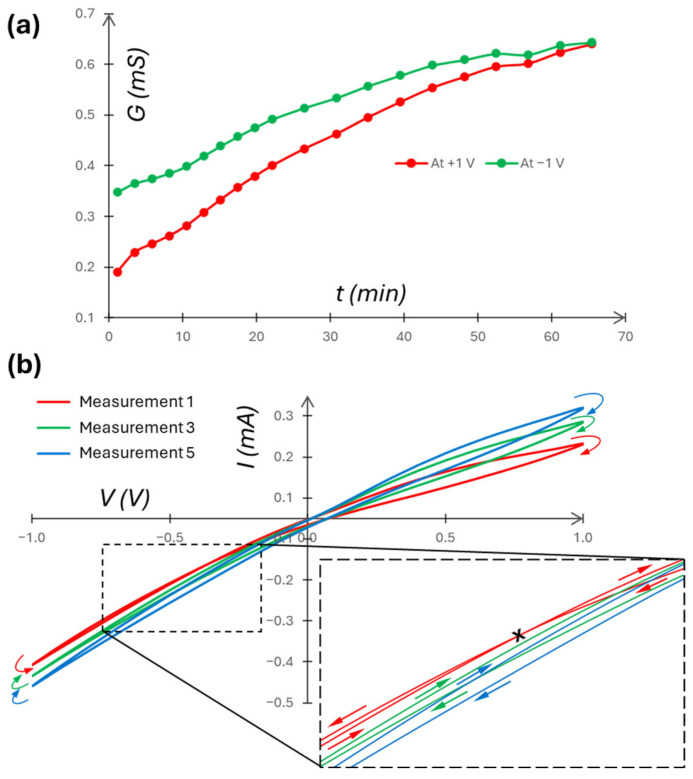
Time-dependent measurements using 0.1|100 mM KCl, an AAO isotropic membrane, and a ±1 V, 0.1 Hz signal. (**a**) Conductance over time measured at both amplitude peaks (at +1 V in red) and troughs (at −1 V in green). (**b**) Multiple measurements over time. Measurement 1 (i.e., cycle 2 of scan 1) begins 1 min 10 s after solution injection, with successive measurements beginning after 2 min, 20 s gaps. The inset magnifies the *I*/*V* self-crossing point of measurement 1 (marked by the x). The arrows indicate scan direction. The arrows indicate scan direction, colored according to their associated measurement.

Because the system evolves over time as the KCl concentration in both reservoirs mixes, first measurements are always taken 1 min after the system is set up (that is, after the *c_L_* and *c_H_* solutions are injected to opposite sides of the membrane interface). To track system evolution over time, a typical measurement consists of a consecutive series of 10 identical voltage-sweeping scans taken with 2 min gaps between them. There are two cycles in each scan. With the exception of Figure 6b, all data in the *I*/*V* plots of this section show the results of the second cycle of the first scan (see Figure S6, Section S2). The duration of the time gaps was chosen to track the state of the device as its electrolyte concentration polarity decreased (which is redundantly long to account for the different speeds of the approach to equilibrium for different membrane types and *c_H_* values). The number of scans was chosen to verify that the applied signal would not create non-reversible effects in the device over time besides the concentration asymmetry (e.g., permanently altering the channel surface chemistry).

As seen in Figure 6a, the overall conductivity of the system begins to increase immediately after the *c_H_* and *c_L_* solutions are allowed to mix (at initial time *t* = 0); i.e., large composite fluidic memristors may have limited memory time, which is a characteristic of large neurons [39,40]. The mixing alters the intended asymmetry of the system and introduces time-varying *R_EE_*_1,2_ and *C_p_* components in the circuit (Figure 4b). As a result, the large composite memristor is intrinsically dynamic, which is expected to exhibit behaviors different from single-pore counterparts. Furthermore, different measurement scan frequencies require different spans of time to complete, and corresponding time-varying *R_EE_*_1*,*2_ and *C_p_* will be different. Nevertheless, overall, robust and reproducible measurements were achieved, i.e., repeated measurements with identical setups yielded the same *I*/*V* results for similar applied signals.

Figure 6b is a typical trajectory of the *I*/*V* relationship over multiple scans (over time). It also shows the effects of solution mixing on the dynamic behavior of the composite memristor system. A transition from self-crossing (type I) (due to *C*_1_*M*_2_ coupling [61] (p. 6462)) to a non-self-crossing (type II) memristive state, analogous to *C*_2_*M*_3_ coupling in [61], is displayed. The time-varying *R_EE_*_1,2_ and *C_p_* are likely driving the state evolution. However, the detailed transition processes from *C*_1_ (positive capacitance) to *C*_2_ (negative capacitance or inductance) and *M*_2_ to *M*_3_ are not clear. In Figure 6 of [63] (p. 13), a similar transition in an FTO/PEDOT:PSS/CH3NH3PbBr3/Au memristor device due to applied voltage increase was linked to a transition from capacitive to inductive hysteresis [63]. In [28], the 100 Hz frequency operation caused non-self-crossing hysteresis of nanopore memristors, which showed self-crossing memristor behaviors at lower frequencies [28].

**Figure 7:**
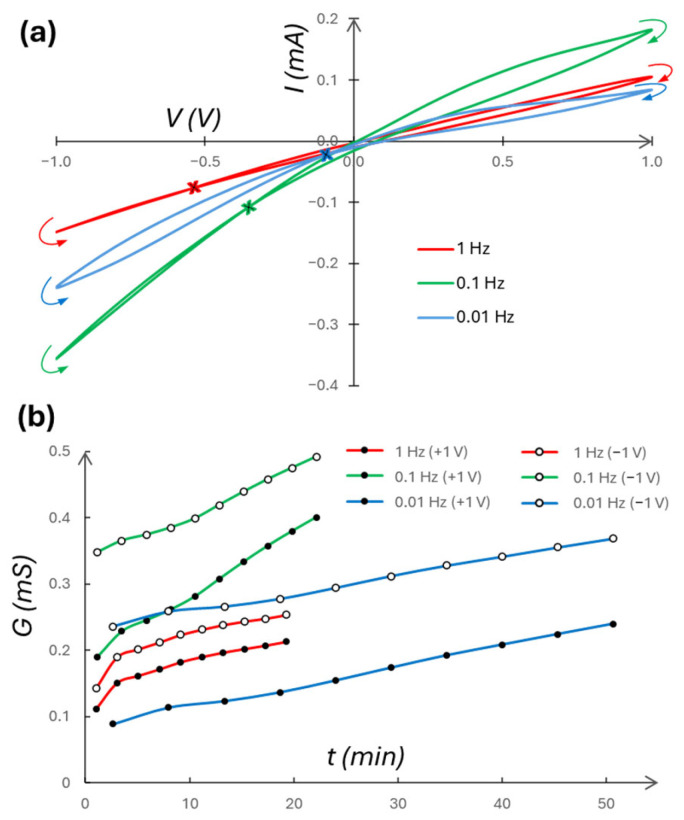
(**a**) *I*/*V* plot of varying signal frequencies. Scan directions are indicated by arrows and self-crossing points are indicated by an “x”, both colored according to their associated measurement. (**b**) Conductance taken at the peaks and troughs of the voltage signal, measured over time for each of the three frequencies. Measurement number is constant for comparable statistics. Both use 0.1|100 mM KCl, an AAO Isotropic membrane, and a 1 V signal amplitude.

Figure 7a shows stronger capacitive coupling effects (e.g., *C*_1_*M*_2_ in [61] (p. 6462)) for higher frequencies. At 0.01 Hz, the self-crossing *I*/*V* hysteresis loop is more symmetric, implying weaker capacitive coupling effects, though the self-crossing point deviates from (*I* = 0, *V* = 0). Further, for 1, 0.1, and 0.01 Hz, the memory window [64] at read voltages (−0.8 V, +0.5 V) are, respectively, (1.02, 1.28), (1.03, 1.50), and (1.15, 1.77). Figure 7b shows the conductance variation over time of the composite memristor at different frequencies. There is no monotonic frequency dependence, likely due to the frequency-dependent capacitance effects in the composite memristor. In Figure 7b, the degree of asymmetry of electrolyte concentration can be estimated by the ICR factor—the ratio between the peak reverse (at −1 V) and peak forward (at +1 V) currents, |I_R_/I_F_|. E.g., for the first scans (Figure 7a) with frequencies 1, 0.1, and 0.01 Hz, the respective ICR factors are 1.41, 1.96, and 2.83. We also note the time dependence of this parameter. At around t = 20 min (Figure 7b), the solutions have been sufficiently mixed to lower the ICR factor to 1.27 using 0.1 Hz. Using 0.01 Hz at the same point in time results in an ICR factor of 2.08 with an *I*/*V* self-crossing point still occurring at around −400 mV. The time-dependent degree of asymmetry indicates a noticeable role of the frequency of *V* in electrolyte transport through the membrane nanopores or nanochannels.

**Figure 8:**
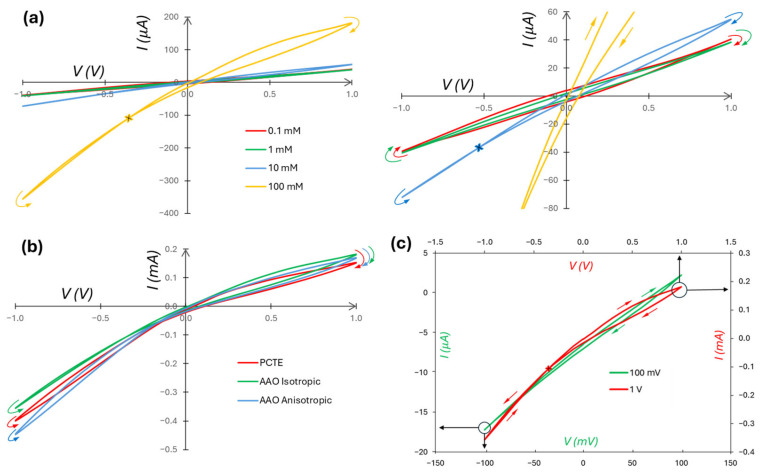
*I*/*V* dependence using different device parameters. In all cases, a voltage of ±1 V, 0.1 Hz is applied. Arrows indicate scan direction and an “x” indicates a self-crossing point, both colored according to the corresponding measurement. (**a**) (Left) varying *c_H_* while holding *c_L_* constant at 0.1 mM KCl, and (right) same plot with a truncated y-axis to show more clearly the 0.1|0.1 mM, 0.1|1 mM, and 0.1|10 mM measurements. (**b**) Varying the membrane type; all measurements trend in the same direction, and all have a self-crossing point (not marked) in the third quadrant. (**c**) Varying the applied voltage amplitude.

To examine the dependence of ICR and memristive features on the degree of asymmetry in electrolyte concentrations, we vary the *c_H_* concentration from 0.1 to 100 mM while holding the *c_L_* concentration constant at 0.1 mM (Figure 8a). For a *c_H_* of 0.1, 1, 10, and 100 mM, the respective ICR factors are 0.98, 1.05, 1.32, and 1.96. As expected, larger concentration asymmetry induces larger rectification, i.e., nonlinearity, when all else is held constant. Figure 8b is the comparative results of using PCTE, AAO isotropic, and AAO anisotropic membranes. Summarized in Table 1, each membrane has a unique pore size, channel length, pore density, and surface charge density, all of which are expected to contribute to ICR and overall conductivity. Quantitative comparisons between the memristors require isolation of the contribution of each factor and precise modeling of the parasitic capacitance and time-varying resistance. Such information is not currently available. Nevertheless, the substantial difference between the hysteretic area in the reverse-current loops of the AAO isotropic (20 nm pore diameter) and PCTE and AAO anisotropic (10- and 5 nm pore diameters, respectively) membranes agrees with the expectation that less confined channels yield weaker memristive performance. ICR and memristive behavior can also be modulated by the applied voltage, as shown in Figure 8c, as a higher voltage leads to more substantial differences between the depleted and enriched states of the channels [25]. Applying a 100 mV signal amplitude results in a rectification ratio of about 1.1, as opposed to 1.96 when using 1 V.

**Figure 9:**
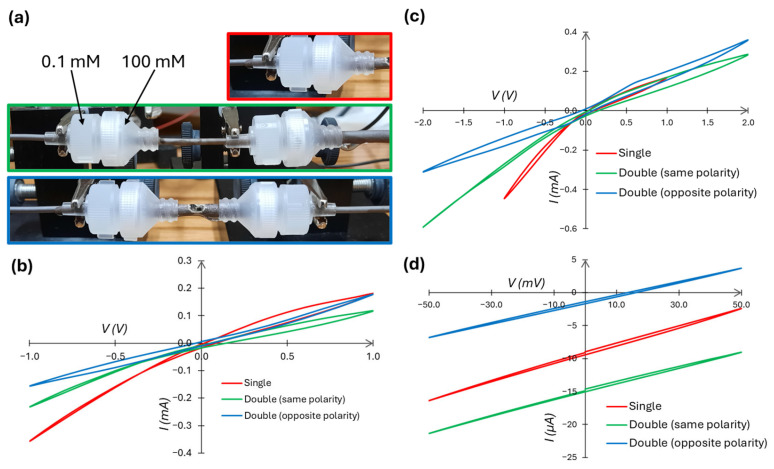
*I*/*V* measurements using two membrane devices in series with a single membrane device as reference. All devices use 0.1|100 mM KCl and a 0.1 Hz signal. (**a**) Photographs of the measurement setup: (red/top) a single memristor, (green/middle) two memristors in series, both with the orientation of the single device, and (blue/bottom) two devices in series, where the second is in the opposite orientation as the first. (**b**) Measurements of all devices using AAO Isotropic membranes in each holder. (**c**) Measurements of all devices, with AAO Anisotropic membranes in each holder and using a signal amplitude of 2 V for both double-membrane devices and a 1 V amplitude for the single-membrane device. (**d**) Measurements of all devices, with AAO Anisotropic membranes in each holder and using a signal amplitude of 50 mV.

A second type of composite memristor, i.e., connecting two large memristors in series, is measured and presented in Figure 9. Such circuits have been analyzed theoretically and computationally [47,48,49,65], but not experimentally with fluidic memristors. Analogous to diodes, a series combination of two identical systems with the same orientation/polarity (Figure 9a, middle) is expected to result in a doubling of the “on” and “off” state resistances in both respective directions; indeed, this is approximately what we observe (Figure 9b–d). A series combination of two memristors with opposite polarities (the bottom image in Figure 9a) results in a nearly symmetric current response—a rectification ratio of 0.87 (Figure 9b) and 0.86 (Figure 9c). Note also that the memristive self-crossing point (seen in the single and double (same polarity) measurements of Figure 9b,c) disappears when the polarities are opposed.

All three configurations of Figure 9a have a unique zero-current potential, as seen more clearly when the sweeping voltage is limited to 50 mV (Figure 9d). This is attributed to the membrane potential characterized by the fixed-charge theory of Teorell [66]. The potential reduces when the systems are oriented with opposite polarity, which is expected as the working (left-hand-side) and counter (right-hand-side) electrodes are submerged in the same concentration solution. Hence, there is little difference in the electrode potentials (which we attribute to small measurement timing errors of the two *c_H_* solution injections), and the reversal potentials [57] are equal and opposite. When the systems are oriented with the same polarity, there is a larger difference between the electrode potentials (as with the single-membrane setup), and the reversal potentials are combined.

## 4. Discussion and Conclusions

The obtained *I*/*V* relationships in Figure 5c and Figure 9b,c show clear nonlinear and memristive behaviors of extra-large composite memristors built with commercial nanopore membranes. The results qualitatively agree with the simulation results of a single truncated nanochannel in Section S1. In general, such a nanochannel with asymmetric electrolytes induces nonlinear ionic currents when applied voltage is slowly tuned and the electrolyte Debye length is comparable to the critical dimension of the structures, such as pore diameters. When voltage sweeping frequency is increased and ion motion is not in sync, memristive effects are recorded. At even higher frequencies, ions are too slow to respond, and the devices behave as resistors (Figure 7a) [26].

The measured mA-level currents indicate strong driving capabilities, which also depend on electrolyte concentration asymmetry, voltage sweeping frequency, voltage level, and pore size. It is estimated that each isotropic AAO and PCTE 10–20 nm pore carries a current of 2 pA, approximately, at 1 V applied voltage (Figure 8). The current is approximately 10 times lower than the results from a COMSOL Multiphysics 5.6 simulation shown in Figure S3, Section S1. Significant current difference is expected due to the truncated channel length in Figure S3a and voltage division by *R_EE_*_1_ and *R_EE_*_2_ in Figure 4b. Compared with other reported results on nanopore memristors in [27,28,29], the measured current in this work is approximately 10^4^ times lower since 300 pores [28,29] in parallel would yield a current of 600 pA only. Further work is needed to determine the factors that caused such large current differences.

The measured voltage values in Figure 5, Figure 6, Figure 7, Figure 8 and Figure 9 include voltage drops across all the components in Figure 4b. The actual voltage across the membrane can only be obtained after removing the parasitic impedance contributions of *R*_EE1_, *R*_EE2_, and *C*_p_ in Figure 4b. A de-embedding operation, including measuring the system in Figure 2b with and without the membrane, is needed. For instance, EIS measurements of the symmetric-concentration (0.1|0.1 mM and 100|100 mM) systems without a membrane can help estimate the *R*_EE_ for each side (*c_L_* and *c_H_*) of the system. Subtracting those values from the 0.1|100 mM system with the membrane at the moment of system initialization would retrieve the membrane resistance. The estimated *R*_EE_ values and total resistance are expected to show much stronger nonlinear and memristive behaviors of the composite memristor.

A few approaches can be explored to slow the rapid change in solution concentration asymmetry *c_H_* versus *c_L_*. The first is to use relatively fewer pores. For instance, commercial membranes with 10 times lower porosity are available, and most of the pores can be blocked (Figure 1b). Thus, a 0.1 mm diameter opening area and a 1% porosity can reduce the number of pores to ~5 × 10^6^ for the 5 nm pores (Table 1). Secondly, larger *c_L_* and *c_H_* reservoirs can be used. Automatic system operation that can enable immediate current measurements after solution injection can also aid experimental reliability. Overall, a factor of 10^3^–10^4^ improvement in addressing the electrolyte diffusion/mixing challenge can be achieved, which is expected to enable the de-embedding operation discussed above. As a result, accurate and even more reproducible measurements can be conducted to uncover the electrochemical and electro-physical processes in the extra-large composite memristors. The results will enable accurate electrical modelling of large fluidic memristors, and more complicated multi-membrane circuitry, exhibited in Figure 9, can be constructed. Therefore, exploiting commercial nanopore membranes is an effective approach for fluidic memristor studies, especially for large memristors.

The composite fluidic memristors are volatile. The obtained ICR values and relatively small loop areas indicate low ON/OFF resistance ratio and short memory time, though both are reasonably close to reported results of single- or 300-nanopore fluidic devices [22,28,29] and similar to the performance of the solid-state volatile memristors [67]. Such memristive properties have not been widely reported or explored despite their significance in certain important applications. For example, short memory and low ON/OFF ratios are crucial to enable Purkinje cells for the immediate processing of sensory inputs and motor errors during motor tasks [68,69,70,71]. The observed behavior of nanopore arrays may be of interest to study devices analogous to giant synapses, such as neuromuscular junctions, which release a vast number of neurotransmitters simultaneously [72,73,74]. Furthermore, the frequency, voltage, and material-dependent ion transport process may shed light on the development of electrical-field-assisted ion-selective-membrane filtration science and techniques [75,76]. However, the use of the proposed nanopore array does not address the problems of current fluidic memristors in scaling and CMOS compatibility as well as achieving ion selectivity and active or non-volatile (or threshold-based [77]) operations. All of these, as well as high ON/OFF resistance ratio and long-term fluidic memory, will need new approaches.

In summary, nanopore array-based large memristors were built and measured with different membrane materials, electrolyte types and concentrations, and applied voltage levels and sweep frequencies. The surface charge of nanopores was experimentally characterized. For asymmetric electrolyte concentrations, the obtained *I*/*V* results show clear nonlinear behavior, which was qualitatively supported with COMSOL simulations (Section S1). Measured device conductance was dependent on time and voltage sweeping frequency. Device memristive behavior was also dependent on measurement time, voltage sweeping frequency, voltage level, and electrolyte concentration. Additionally, memristors in series with the same or opposite polarity were characterized. Nevertheless, further work is needed to enable automatic *I*/*V* measurement and accurately de-embed the parasitic effects of the measurement circuits. The simple nanopore membrane provides a flexible and adaptable model system to investigate fluidic memristors, especially memristor dynamics.

## Figures and Tables

**Table 1:** Membrane and channel/pore dimensions, including those respective to the membrane‘s active layer (AL) and support layer (SL) if applicable. All membranes are 13 mm in diameter. Abbreviations “Iso.”, “Ani.”, and “por.” denote “Isotropic”, “Anisotropic”, and “porosity”, respectively. $N_{AL}$ and $N_{SL}$ are the total number of pores on the AL and SL, respectively.

Membrane	$D_{AL}$ [nm]	$L_{AL}$ [μm]	$D_{SL}$ [nm]	$L_{SL}$ [μm]	AL por.	SL por.	$N_{AL}$	$N_{SL}$
PCTE	10	6±0.6	N/A	N/A	0.05%	N/A	$7.98\times 10^{8}$	N/A
AAO Iso.	20	50±2	N/A	N/A	15±2%	N/A	$6.63\times 10^{10}$	N/A
AAO Ani.	5	5±0.5	100±20	59±2	12±2%	20–25%	$8.11\times 10^{11}$	$3.80\times 10^{9}$

## Data Availability

The original data presented in the study are openly available in FigShare at https://doi.org/10.6084/m9.figshare.29661896.

## Supplementary Materials

The following supporting information can be downloaded at: https://www.mdpi.com/article/10.3390/mi16080882/s1, Figure S1: Schematic of a nanochannel and the experimental apparatus (discussed in greater detail); Figure S2: An example EIS measurement to characterize the nanochannels; Figure S3: COMSOL simulation model and results; Figure S4: Simulated ICR/channel length results; Figure S5: Simulated *I*/*V* data for channels of different radii; Figure S6: Representation of the applied potential waveform; Figure S7: *I*/*V* measurements using different electrolyte species.
