# Supplementary material for: Composite Behavior of Nanopore Array Large Memristors
Source: Micromachines (Basel). 2025 Jul 29;16(8):882. doi: 10.3390/mi16080882 (PMC12388671; doi:10.3390/mi16080882)
Supplement: Supplementary file 1 [file micromachines-16-00882-s001.zip › micromachines-3784646-supplementary.pdf]

*Article*

# Composite Behavior of Nanopore Array Large Memristors

**Ian Reistroffer [1,2], Jaden Tolbert [1], Jeffrey Osterberg [1,3] and Pingshan Wang [1,*]**

[1] Holcombe Department of Electrical and Computer Engineering, Clemson University, Clemson, SC 29634, USA; ianreistroffer0@gmail.com (I.R.); tolber3@g.clemson.edu (J.T.); jeff_osterberg@hotmail.com (J.O.)
[2] Department of Physics, South Dakota School of Mines and Technology, Rapid City, SD 57701, USA
[3] Sandia National Laboratories, Albuquerque, NM 87123, USA
* Correspondence: pwang@clemson.edu

## 1. Surface Charge Extraction and COMSOL Simulation of Current Rectification

To help understand the behavior of the nanopore array memristor, we measure the surface charge density of the nanochannel walls and simulate the expected rectification behavior using COMSOL Multiphysics. Due to computational limitations and uncertainties of the nanopore geometry, the simulation results cannot be directly extrapolated to predict the results of the actual measurements. However, an abstracted version of the real setup shows that significant rectification is enabled with a single nanochannel with surface properties similar to those in our commercial membranes. Therefore, we present the measurement of the surface charge density and the following simulation procedures as motivation for conducting real ICR measurements and as a guide for future studies.

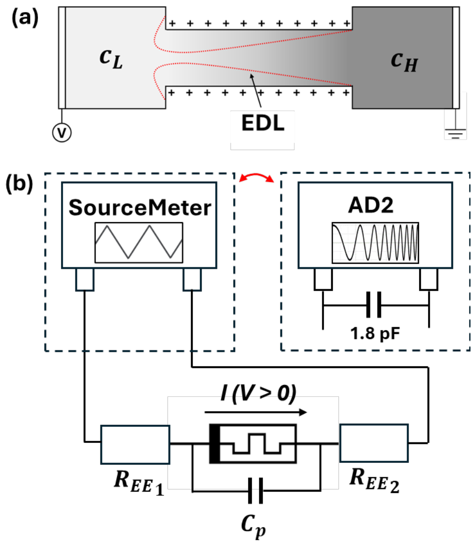


**Figure S1. (a)** Schematic representation of a single nanochannel connecting reservoirs of $c_L$ solution (light grey, low concentration) and $c_H$ solution (dark grey, high concentration). The EDL is represented in the red enclosure, corresponding to an enrichment of anions that have migrated toward the positively charged channel walls. **(b)** A diagram of the electrical measurement circuit. Using the AD2, a parasitic capacitance of 1.8 pF is in parallel with the system. $C_p$ is parasitic capacitance across the nanopore array. $R_{EE}$ is the resistance of the electrode-electrolyte interfaces.

The two electrode-electrolyte interfaces (Figure S1b) contribute parasitic resistance to the nanochannel array [1]. The interface is typically modeled as a resistor (charge-transfer resistance) and capacitor (double layer capacitance) in parallel, together in series with another resistor (solution resistance) [2,3]. However, a 1.8-pF parasitic capacitance in our AD2 device (in parallel with the fluidic system) and the solution resistance limit EIS signal frequencies to the MHz range for analysis. Thus, in this study we consider the electrode-electrolyte interfaces as a single resistor, $R_{EE}$. To measure $R_{EE}$, we apply EIS with a sinusoidal voltage swept over a range of frequencies to obtain the real and imaginary impedance components. The Nyquist plot features (negative imaginary impedance as a function of real impedance) are then matched to those of well-established electro-fluidic models to determine $R_{EE}$ [4]. An example of one such Nyquist plot is shown in Figure S2.

To measure the nanochannel surface charge density $\sigma_s$, the following model is used (with terms corresponding to Figure S1b) [5,6].

$$R_{tot} = \{R_{EE_1} + R_{EE_2}\} + \left\{N_{AL}\left[\frac{\pi r_{AL}^2}{L_{AL}}\left(10^3(\mu_K + \mu_{Cl})c_B N_A q_e + \frac{2\mu_K \sigma_s}{r_{AL}}\right)\right]\right\}^{-1}$$
$$+ \left\{N_{SL}\left[\frac{\pi r_{SL}^2}{L_{SL}}\left(10^3(\mu_K + \mu_{Cl})c_B N_A q_e + \frac{2\mu_K \sigma_s}{r_{SL}}\right)\right]\right\}^{-1}$$

The first bracketed term represents the electrode-electrolyte interface resistance for electrode 1 ($R_{EE1}$) and electrode 2 ($R_{EE_2}$), the sum of which we refer to as $R_{EE}$. The second term represents the resistance of the array of nanopores in the active layer (AL). The third term is that of the support layer (SL), which is only non-zero for the AAO anisotropic membrane (see Table 1). $N$, $r$, and $L$ are, respectively, the number of effective pores, the radius of each pore, and the length of each pore for the indicated membrane layer (AL, SL). Mobilities $\mu_K$ and $\mu_{Cl}$ are for $K^+$ and $Cl^-$ ions ($\mu_K = 7.619\times10^{-8}$ m$^2$ V$^{-1}$s$^{-1}$ and $\mu_{Cl} = 7.912\times10^{-8}$ m$^2$ V$^{-1}$s$^{-1}$) [7]. $N_A$ is Avogadro's number, and $q_e$ is the elementary charge. We approximate the effective number of pores to be those normal to the cross-section of the 2-mm electrode. Fitting measured real impedance to this model allows us to determine the surface charge density $\sigma_s$ of the channel walls.

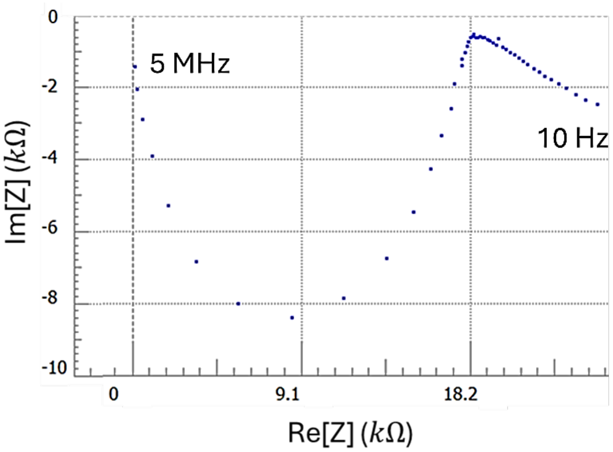


**Figure S2.** An example EIS measurement, in this case using 0.1-mM $CaCl_2$ with no membrane present between the electrodes. Frequency increases from right (10 Hz) to left (5 MHz).

We use the measured $\sigma_s$ to simulate a nanopore system in COMSOL Multiphysics in steady state. Due to computational capacity limitations, only a single nanochannel with a truncated length is examined. Figure S3 shows an example corresponding to the AAO anisotropic membrane, with $\sigma_s = 7.5$ mC m$^{-2}$, similar in magnitude to that found by [8]. The channel in Figure S3a has a length of 50 nm and diameter of 5 nm. The low- and high-concentration reservoirs are set to 0.1 mM and 100 mM KCl, respectively. A voltage from -1 to +1 V in steps of 0.1 V is applied. Rectification is seen in the $I/V$ plot of Figure S3b. The governing equations with boundary conditions are solved using MUltifrontal Massively Parallel sparse direct Solver (MUMPS) [7]. The results indicate that the nanoscale channels created by the pores in our membranes can significantly rectify the current through them. However, there are $10^7$ – $10^{10}$ channels in parallel in our real membranes (Table 1), a composite nanopore device that itself is in series with parasitic resistance ($R_{EE}$) on the order of a few kilo-ohms. To minimize $R_{EE}$ (Figure S1), we bring the electrodes as close as possible (~10 μm) to the membrane surface.

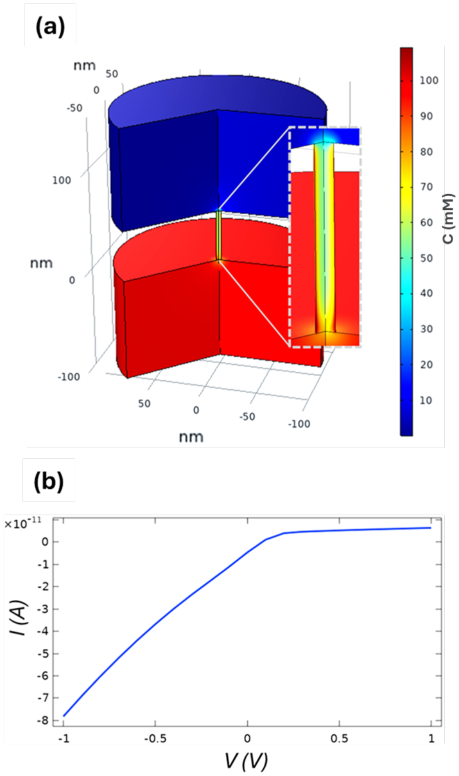


**Figure S3. (a)** A 3-D schematic of the simulated fluidic system and its boundary conditions. The color gradient shows the KCl concentration profile in mM when a -1 V bias is applied (at the top). **(b)** An example *I*/*V* result of applying a half-cycle linear voltage sweep. Potential is applied at the top face of the low-concentration reservoir (in blue in (a)) from -1 to +1 V, the resultant ion flux of which is measured through the bottom face of the high-concentration reservoir (in red in (a)) acting as ground, which is then converted into current in amps.

Our simulation setup was further used to explore the mechanism behind the rectification phenomenon. The mechanism expected to control the ICR of a system such as ours is the rate of cation and anion transport into and out of the channel [7]. When the overall ion transport into the channel is greater than the current out of the channel, ions accumulate in the channel and overall current increases. When ion transport into the channel is less than current out of the channel, ions deplete from the channel and overall current decreases. Consider Figure S1a, where the wall charge is positive. The "on" state occurs when a negative potential is applied at the electrode. In that case, anions traveling from left to right enter the channel with a larger current than they exit due to the enriched EDL region on the left-hand side. Additionally, cations traveling from right to left enter the channel with a larger current than they exit due to the higher screening of the positive-potential EDL on the right-hand side. If the contribution of these two charge-transport mechanisms to overall current asymmetry is imbalanced, then determining which has a larger effect can help guide parameter decisions of real measurements.

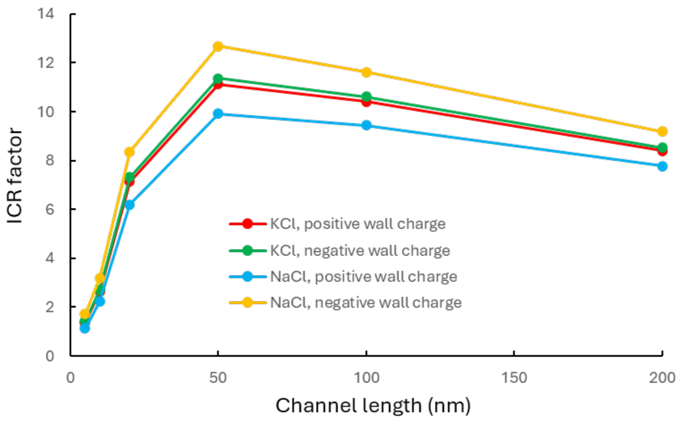


**Figure S4.** Simulated rectification factors of single nanochannels with positive and negative surface charge densities (±7.5 mC $m^{-2}$) and varying ionic species over channel length. Current measurements are taken at +/- 1 V. In all cases, the reservoir on the side of the applied potential contains the low-concentration (0.1-mM) solution and the opposite reservoir contains the high-concentration (100-mM) solution.

We simulate current rectification with KCl and NaCl ($\mu_{Na}$ = 5.20 × $10^{-8}$ $m^2$ $V^{-1}s^{-1}$) with positive and negative wall charges (Figure S4). We find that when the ions of the electrolyte have different diffusion coefficients in water (as is commonplace), rectification can be increased when the wall charge has the same electric polarity as the ion with the larger diffusion coefficient. We show that this relationship is consistent with a range of nanochannel lengths from 5 nm to 200 nm. It seems therefore that the counter-ion current is the dominant source of asymmetry, and that the effect can be amplified with a larger divergence in electrolyte mobilities, as seen in the different rectification ratios when KCl versus NaCl electrolytes are used. In all cases, the simulated rectification decreases as bulk solution resistance increases with channel length (in effect adding a larger resistor in series with a diode-like component).

Computation time increases with channel length, thereby restricting parallelity between our system and the simulation. Further challenges arise in consideration of electrode dimensions. Our real electrodes are 2 mm (on one side) and 4 mm (on the opposite side) in diameter and brought to within 10 μm of the membrane surface. Under these circumstances, the radial propagation of the electric field at the edges of the electrodes needs to be considered. I.e., ion transport will be different through pores more distant from the electrode surface than those directly in front of the electrode. In the simulation, the electrodes (the planes used to measure ion flux) are larger in area than the single nanopore by a factor of 10.

We also simulate the effect of changing the radius of a given channel. In Figure S5, the same 50-nm length channel with asymmetric KCl solution is scanned from -1 to +1 V for a radius of 2.5 nm (like our AAO anisotropic membrane) and 5 nm (like our PCTE membrane). The resulting ICR factor is 11.1 for the

2.5-nm radius channel and 4.64 for the 5-nm radius channel. This is consistent with our expectation that increasing the confinement of the channel will increase overall rectification. We do not see a comparable relationship in our measurements of the PCTE and AAO anisotropic membranes This may be attributable to the differences in pore count or slight differences in length.

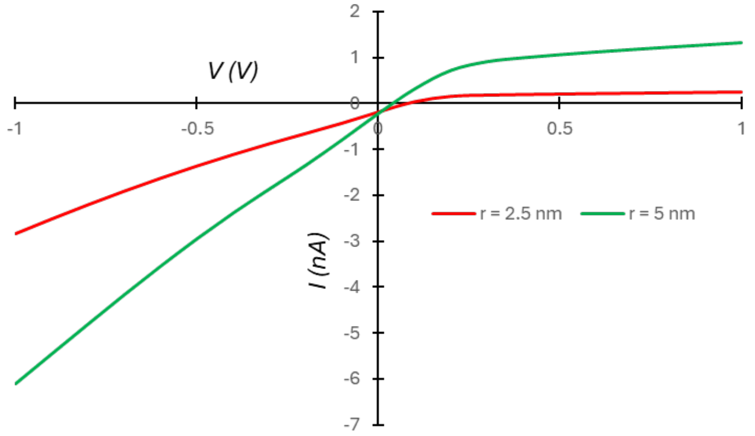


**Figure S5.** Simulated *I*/*V* curves for 50-nm-long channels of different radii. The reservoir on the side of the applied potential contains 0.1-mM solution; the reservoir on the opposite side contains 100-mM solution, both KCl.

## 2. Voltage sweep and data acquisition procedures

The electrodes supply a voltage (either ±100 mV or ±1 V) across the system. The voltage is delivered as a triangle waveform (Figure S6) with a sweeping frequency of 0.01, 0.1, or 1 Hz by a Keithley 2612 SourceMeter (Figure S1b). The waveform is produced by incrementing a staircase voltage over 40 steps and is applied for 2 consecutive cycles. The output current is measured at the end of each step. Because the system evolves over time as the concentration in both reservoirs mix, the beginning ($t = 0$ in Figure S6) of the *I*/*V* measurement process is always initiated 1 minute after the system is set up (i.e., after the low- and high-concentration solutions are injected and allowed to flow across the membrane interface), and a series of 10 identical consecutive scans are taken with 2-minute gaps between them. In other words, in one complete "set" of measurements, 10 voltage-sweeping scans are performed, each scan containing two cycles. Figure S6 shows an example of a first scan when a 1-V amplitude, 0.1-Hz signal is applied. With the exception of Figure 6b, the *I*/*V* plots in Section 3 of the main article always show the result of the second cycle of the first scan in the measurement set (e.g., from $t = 10$ to $t = 20$ sec in Figure S6).

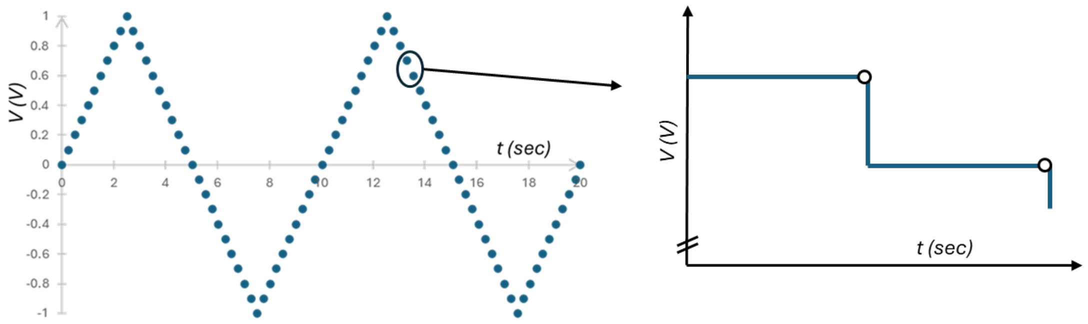


**Figure S6.** A typical applied voltage signal (here with a 1-V amplitude and 0.1-Hz frequency) used in a typical *I*/*V* measurement. The inset on the right zooms in on two descending potential steps, with hollow circles marking where the current measurement at each step is taken.

Careful and frequent re-conditioning of the Ag/AgCl electrodes can also improve signal reproducibility. For example, the results of our variable electrolyte species measurements (Figure S7) show a double-crossing *I*/*V* curve using $CaCl_2$ during its first scan. Such an *I*/*V* curve was shown in simulation in [9] and $C_2M_4$ coupling in [10]. Double-crossing was also observed in nanopore fluidic memristors with $CaCl_2$ in [11]. However, in this work, the relative values of the overall measured conductance do not agree with approximations based solely on electrolyte conductivity. In addition to possible coupling between parasitic capacitance, as shown in [10], the measurements may have been affected by a prior overuse of the electrodes, a period during which they were subjected to the experimental conditions of the 1-V, 0.01-Hz scan (Figure 7). This measurement could have, over subsequent scans, depleted the active surface on the silver metal (an over-accumulation or depletion of the AgCl layer) which would have acted parasitically to the overall current [12]. The issue was later resolved by sanding down the electrode surface, rinsing it with DI water, and submerging it in 1-M NaClO for 30 minutes after each use.

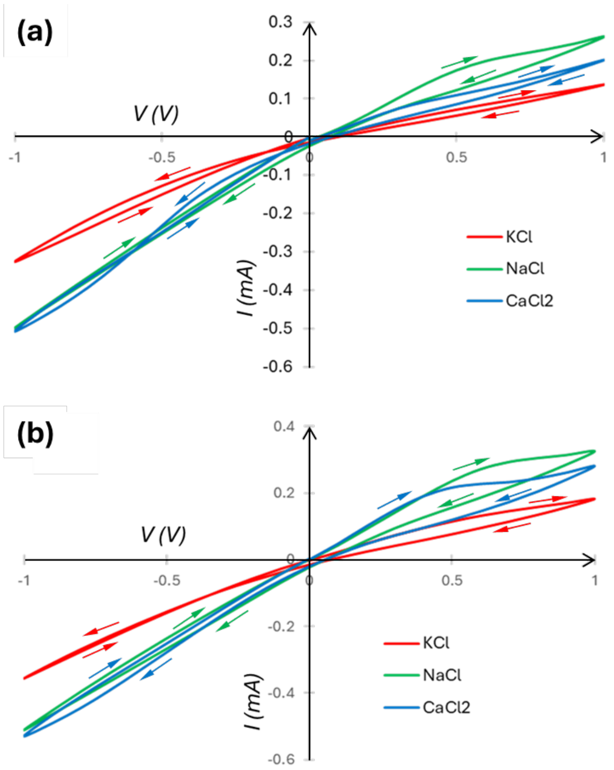


**Figure S7.** *I*/*V* measurements from **(a)** scan 1 and **(b)** scan 2, using 0.1 ∣ 100 mM variable solution and a 1-V, 0.1-Hz voltage.

## References


1. Nasrollaholhosseini, S.H.; Mercier, J.; Fischer, G.; Besio, W.G. Electrode–Electrolyte Interface Modeling and Impedance Characterizing of Tripolar Concentric Ring Electrode. *IEEE Transactions on Biomedical Engineering* **2019**, *66*, 2897-2905, doi:10.1109/tbme.2019.2897935.
2. Wu, J. Understanding the Electric Double-Layer Structure, Capacitance, and Charging Dynamics. *Chemical Reviews* **2022**, *122*, 10821-10859, doi:10.1021/acs.chemrev.2c00097.
3. Sánchez, Y.P.; Santos, A.; Bueno, P.R. Quantum Mechanical Meaning of the Charge Transfer Resistance. *The Journal of Physical Chemistry C* **2022**, *126*, 3151-3162, doi:10.1021/acs.jpcc.1c07801.
4. Mei, B.-A.; Munteshari, O.; Lau, J.; Dunn, B.; Pilon, L. Physical Interpretations of Nyquist Plots for EDLC Electrodes and Devices. *The Journal of Physical Chemistry C* **2018**, *122*, 194-206, doi:10.1021/acs.jpcc.7b10582.
5. Yeh, L.-H.; Zhang, M.; Qian, S. Ion Transport in a pH-Regulated Nanopore. *Analytical Chemistry* **2013**, *85*, 7527-7534, doi:10.1021/ac401536g.
6. Martins, D.; Chu, V.; Conde, J.P. The effect of the surface functionalization and the electrolyte concentration on the electrical conductance of silica nanochannels. *Biomicrofluidics* **2013**, *7*, doi:10.1063/1.4811277.
7. Cheng, L.-J.; Guo, L.J. Rectified Ion Transport through Concentration Gradient in Homogeneous Silica Nanochannels. *Nano Letters* **2007**, *7*, 3165-3171, doi:10.1021/nl071770c.

8. Hiller, D.; Tröger, D.; Grube, M.; König, D.; Mikolajick, T. The negative fixed charge of atomic layer deposited aluminium oxide—a two-dimensional SiO<sub>2</sub>/AlO <sub>x</sub> interface effect. *Journal of Physics D: Applied Physics* **2021**, *54*, 275304, doi:10.1088/1361-6463/abf675.
9. Robin, P.; Emmerich, T.; Ismail, A.; Niguès, A.; You, Y.; Nam, G.-H.; Keerthi, A.; Siria, A.; Geim, A.K.; Radha, B.; et al. Long-term memory and synapse-like dynamics in two-dimensional nanofluidic channels. *Science* **2023**, *379*, 161-167, doi:doi:10.1126/science.adc9931.
10. Sun, B.; Chen, Y.; Xiao, M.; Zhou, G.; Ranjan, S.; Hou, W.; Zhu, X.; Zhao, Y.; Redfern, S.A.; Zhou, Y.N. A unified capacitive-coupled memristive model for the nonpinched current–voltage hysteresis loop. *Nano letters* **2019**, *19*, 6461-6465.
11. Ramirez, P.; Portillo, S.; Cervera, J.; Nasir, S.; Ali, M.; Ensinger, W.; Mafe, S. Neuromorphic responses of nanofluidic memristors in symmetric and asymmetric ionic solutions. *The Journal of Chemical Physics* **2024**, *160*.
12. Tang, X.; Chen, X.; Mak, P.; Gao, Y.; Vai, M. *Investigation of residual Ag amount into human body using Ag/AgCl electrodes during IBC*; 2016; pp. 1-4.
